# The Endoplasmic Reticulum and Its Contacts: Emerging Roles in Axon Development, Neurotransmission, and Degeneration

**DOI:** 10.1177/10738584231162810

**Published:** 2023-03-24

**Authors:** Marijn Kuijpers, Phuong T. Nguyen, Volker Haucke

**Affiliations:** 1 Donders Institute for Brain, Cognition and Behaviour, Radboud University, Nijmegen, The Netherlands; 2 Leibniz-Forschungsinstitut für Molekulare Pharmakologie (FMP), Berlin, Germany; 3 Department of Biology, Chemistry, Pharmacy, Freie Universität Berlin, Berlin, Germany; 4 Charité – Universitätsmedizin Berlin, Charitéplatz 1, Berlin, Germany

**Keywords:** endoplasmic reticulum, neuron, axon, neurotransmission, neurodegeneration, membrane contact sites, neurodevelopment, calcium, lipids

## Abstract

The neuronal endoplasmic reticulum (ER) consists of a dynamic, tubular network that extends all the way from the soma into dendrites, axons, and synapses. This morphology gives rise to an enormous membrane surface area that, through the presence of tethering proteins, lipid transfer proteins, and ion channels, plays critical roles in local calcium regulation, membrane dynamics, and the supply of ions and lipids to other organelles. Here, we summarize recent advances that highlight the various roles of the neuronal ER in axonal growth, repair, and presynaptic function. We review the variety of contact sites between the ER and other axonal organelles and describe their influence on neurodevelopment and neurotransmission.

## Introduction

**Figure 1.**
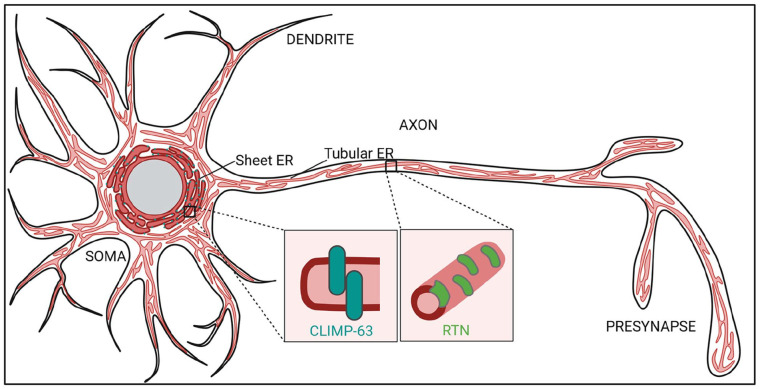
The neuronal endoplasmic reticulum (ER). In neurons, the ER extends throughout all cellular processes. “Rough,” ribosome-studded sheets, as well as associated sheet-inducing protein cytoskeleton-linking membrane protein 63 (CLIMP-63), are enriched in the somatodendritic domain. “Smooth” tubular ER is distributed throughout the soma, dendrites, axons, and pre- and postsynaptic terminals. The term *smooth* indicates the absence of ribosomes on the outer surface. The formation of the tubular ER networks involves ER-shaping proteins such as the reticulons (RTNs).

The endoplasmic reticulum (ER) is a huge, continuous network of membranes (Fig. 1) within cells that serves many important functions. Whereas the ribosome studded “rough” ER (RER) is known for its role in protein synthesis, the tubular or “smooth” ER (SER, devoid of ribosomes) is largely devoted to the biosynthesis and metabolism of lipids and calcium (Ca^2+^) homeostasis. These lipids, proteins, and ions have to be distributed to other membranes at the right time to allow the proper functioning of other organelles and are of key importance for cell signaling. Lipid transport is mediated by the secretory pathway (e.g., vesicular and tubular carriers) and by so-called membrane contact sites (MCSs) formed between the ER and other membranous organelles. MCSs consist of two opposing membranes that communicate through a narrow gap, typically within 10 to 30 nm (Wong and others 2019), and depend on protein-protein and protein-lipid interactions. MCSs may be present constitutively or can form dynamically in response to signaling events or as a consequence of alterations in membrane composition. At MCSs, tethering factors, lipid-transfer proteins, enzymes, and ion channels act synergistically to facilitate the local flow of ions, lipids, and other small molecules. Being the major source of membrane building blocks and the largest membrane-bound organelle in eukaryotic cells, the ER engages in a large number of MCSs with essentially all other organelles, including mitochondria, peroxisomes, lysosomes, endosomes, and the plasma membrane (PM). The ER network is also highly dynamic with sheets or narrow tubules constantly forming, rearranging, and contracting. Although the purpose of these morphological transitions is not fully understood, they can facilitate luminal cargo flow (Holcman and others 2018) in addition to membrane fission (e.g., of mitochondria) (Friedman and others 2011; Wu and others 2018) or budding processes (e.g., lipid droplet biogenesis, ER exit of secretory proteins) (Santinho and others 2020; Stadler and others 2018). Within a single, interconnected ER network, different domains vary greatly in their morphology. Relatively flat areas called ER sheets are often correlated with RER while thin ER tubules are believed to be important for SER linked functions such as lipid biosynthesis and Ca^2+^ release. At the base of shaping these different domains are transmembrane and membrane-associated protein families, including the reticulons (RTN), atlastins (ATL), and receptor expression-enhancing proteins (REEPs) that promote high membrane curvature and fusion, thereby generating and stabilizing ER tubules (Goyal and Blackstone 2013; Shibata and others 2006). The transmembrane protein cytoskeleton-linking membrane protein 63 (CLIMP-63), on the other hand, is mostly associated with RER, possibly functioning as a spacer that maintains ER sheet morphology (Goyal and Blackstone 2013) (Fig. 1). Many of these proteins, such as REEP-1 and CLIMP-63, can also tether the ER to the cytoskeleton and associated motor proteins, thereby modulating ER structure and driving ER network dynamics (Foster and others 2022; Zamponi and others 2022).

**Figure 2.**
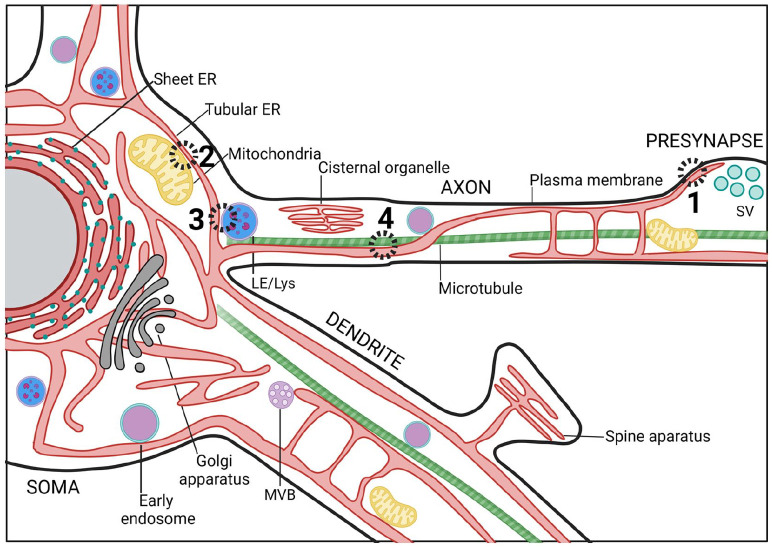
Contact sites between endoplasmic reticulum (ER) and various organelles in neurons. Scheme illustrating the contacts that the ER makes with other cellular compartments throughout soma, dendrites, and axon. (1) ER-plasma membrane contact sites (MCSs) have been proposed to facilitate neurite outgrowth and cellular development by transferring lipids between membranes. Besides, these contact sites can also regulate axonal Ca^2+^ homeostasis to modulate neurotransmission. (2) ER-mitochondria MCSs are critical for mitochondrial Ca^2+^ homeostasis. Absence of ER-mitochondria MCS proteins results in decreased synaptic plasticity and activity. (3) ER contacts with late endosomes (LEs) or lysosomes (Lys) often occur at preaxonal regions and are implicated in LE/Lys fission, maturation, and sorting. (4) Microtubule dynamics are tightly coregulated with ER dynamics. MVB, multivesicular endosome/body; SV, synaptic vesicles.

**Figure 3.**
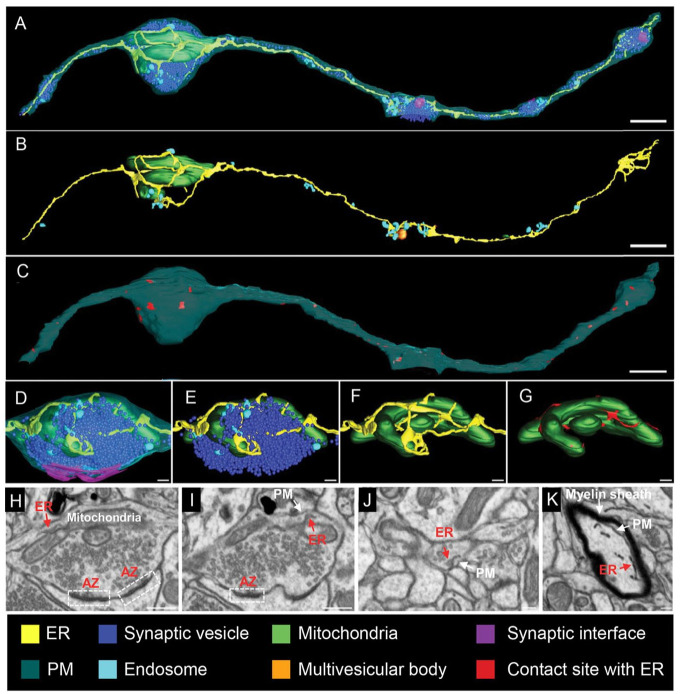
The three-dimensional (3D)–electron microscopy reconstructions and images of axonal endoplasmic reticulum (ER). (A–D) Focused ion beam-scanning electron microscopy (FIB-SEM) is used to map the ER and its connections to other membranes within the axon (nucleus accumbens). The 3D reconstructions show all membranous organelles in (A). In (B), a selective view of the ER (yellow), mitochondria (green), and endosomes (light blue) is shown. In (C), the red color marks areas where the ER contacts the plasma membrane (PM). (D–G) High-magnification views of one of the presynaptic structures of the axonal segment shown in (A). Synaptic vesicles are in dark blue and the synaptic interface in magenta. Red areas in (G) highlight ER-mitochondria contact sites. (H, I) Gallery of micrographs, used to make the presynaptic 3D reconstructions, shows the contacts between presynaptic ER and mitochondria (H) or the PM (I). (J, K) Micrographs illustrating cross sections of axons and indicating contacts between the ER and the PM. Scale bars: 800 nm in A–C; 160 nm in D–G; 400 nm in H and I; 160 nm in J and K. Adapted from Wu and others (2017).

The segregation of different ER subdomains is especially overt in highly polarized cells such as neurons (Fig. 2). Recent developments in cryo-fixation and EM imaging have revealed the fine ultrastructural details of ER subdomains in neurons. These have led to the discovery that the somatodendritic compartment is filled with RER, whereas axons mostly consist of extremely thin (~10–30 nm) smooth ER tubules that run in parallel along the axonal length. At synaptic varicosities, these tubules branch or form small cisternae (Hoffmann and others 2021; Terasaki 2018; Wu and others 2017). ER tubules and cisternae are generally devoid of ER-bound ribosomes, which have been observed only at axonal branch points (Nedozralova and others 2022). The three-dimensional–electron microscopy (3D-EM) and live super-resolution imaging on rodent neuron cultures, brain tissue, and human brain organoids show that thin tubular ER membranes can make contacts with axonal microtubules, mitochondria, endosome-like structures, and the PM (Damenti and others 2021; Foster and others 2022; Hoffmann and others 2021; Wu and others 2017) (Figs. 2 and 3). Other, neuron-specific ER subdomains exist as well. Mature dendritic spines of cortical and hippocampal neurons contain a spine apparatus (i.e., a highly dynamic stack of ribosome-free ER that is continuous with the ER tubules in the dendritic shaft). This spine apparatus plays important roles in Ca^2+^ homeostasis and dendritic spine plasticity (Aloni and others 2021; Dubes and others 2022). A similar ER-based structure called the cisternal organelle can be found in the axon initial segment (AIS) of some central nervous system (CNS) neuron types such as dentate granule cells and cortical principal cells. The AIS is considered the site of action potential generation and plays a role in the sorting of proteins into the axon. The precise function of the cisternal organelle at the AIS, however, is still elusive (Sree and others 2021). Although considered one continuous organelle, fission and fusion events and disconnected ER-like elements have been observed in dendrites (Carter and others 2020; Kucharz and others 2009) and at presynaptic compartments (Wu and others 2017). Whether these ER fragments fulfill specific physiologic roles, such as mobile Ca^2+^ storage or local translation, is unknown.

Considering its many roles, domains, and vast surface area in neurons, it might not come as a surprise that the ER plays a role in a variety of key neuronal functions, including neurite outgrowth and neurotransmission. This notion is strengthened by the fact that mutation in ER morphology and degradation-regulating proteins, including ATL1, ATL3, REEP, and FAM134, are common causes of the motor neuron diseases hereditary spastic paraplegia (HSP) and hereditary sensory and autonomic neuropathy (HSAN). In addition, alterations in ER contact sites have been implicated in multiple neurodegenerative disorders (Ozturk and others 2020). In this review, we discuss recent findings supporting the relevance of the ER to neuronal development and regeneration and to neurotransmission in the mature nervous system. We focus on particular features of the ER—namely, its many contacts with other organelles and their associated roles in lipid and Ca^2+^ homeostasis.

## ER-PM Contact Sites and Lipid Transfer in Development and Regeneration

**Figure 4.**
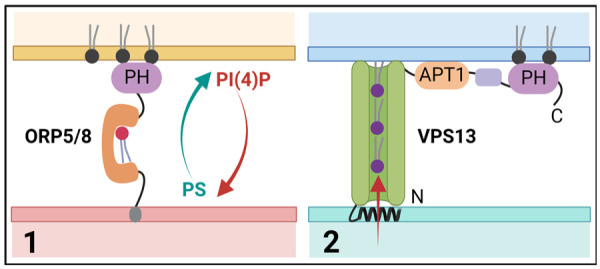
Lipid transport at membrane contact sites. Scheme illustrating different models for lipid transport at endoplasmic reticulum (ER)–plasma membrane (PM) contact sites. (1) Single lipid transport via ORP5/8 at ER-PM contact sites, where they contribute to the exchange of phosophatidylinositol 4-phosphate (PI(4)P) with phosphatidylserine (PS). (2) Bulk lipid transport via VPS13 proteins that contain a lipid-binding APT1 domain. ORP5/8, oxysterol-binding protein (OSBP)–related protein 5 and 8; VPS13, vacuolar protein sorting 13.

The growth of extremely long extensions (i.e., the axon and dendrites) is critical for long-distance communication between neurons. To support their development and repair, massive amounts of membrane components need to be added. Due to their extensive length, vesicular transport in neurons might be insufficient to sustain growth. In contrast, neuronal ER tubules with their vast surface area and roles in nonvesicular lipid transport seem to be perfectly equipped for regulating fast PM growth and regeneration-associated pathways. In agreement with this, tubular ER-shaping proteins, such as atlastins and reticulons, have been found to be enriched in growing and regenerating axons from *Drosophila* and rodent cultures (but not in dendrites), and this enrichment seems to be essential for regeneration and axonal morphology (Farias and others 2019; Rao and others 2016; Zamponi and others 2022). ER-PM contacts are abundant in neurons (Wu and others 2017), where they may function in the replenishment of ER calcium stores and in lipid recycling (Fowler and others 2019). Tether proteins that form MCSs often serve to facilitate lipid transfer between the respective membranes. Several structurally diverse families of ER-localized protein tethers involved in MCS formation have been identified. These include soluble NSF attachment protein receptor (SNARE) proteins (Petkovic and others 2014), VAMP-associated proteins (VAPs), and members of the TMEM protein family. Some of these might also play roles in extending and repairing the neuronal PM. VAPs (i.e., VAP-A and VAP-B) are abundant type II ER membrane proteins involved in MCS formation throughout the ER. VAPs can bind a variety of different proteins, mostly by their FFAT motifs, including kinases, cytoskeleton protein, and ion channels (more about that later). Lipid-transfer proteins are the most well-studied VAP-binding proteins at MCSs and include the oxysterol-binding protein (OSBP)–related proteins (ORPs). ORPs contain a lipid-binding cavity with affinity for different lipids such as oxysterols, cholesterol, phosphatidylserine (PS), and phosphatidylinositol phosphates (PIPs). Humans express 12 ORP homologues that share partially redundant functions and are involved in the transfer of specific lipids at different ER contact sites (Pietrangelo and Ridgway 2018). While most ORPs target the ER by FFAT-mediated VAP interaction, some (e.g., ORP5 and ORP8) have membrane-spanning domains that enable ER localization on its own. A subset of ORPs (ORP3, ORP5, ORP6, and ORP8) contains PH domains that bind phosphoinositides, including phosophatidylinositol 4-phosphate (PI(4)P) and/or phosphatidylinositol 4,5-bisphosphate (PI(4,5)P_2_), and localize them to ER-PM contact sites, where they are primarily involved in the exchange of PS with PI(4)P (Nakatsu and Kawasaki 2021) (Fig. 4). In mouse primary cerebellar granule neurons, ORP6 colocalizes with ORP3 (but not ORP5) at ER-PM contact sites, where it has been suggested to play a role in the countertransport of PI(4)P and PS. Its exact contribution to membrane dynamics and neuronal function is not understood (Mochizuki and others 2018, 2022). Although ORP2 lacks a PH domain, it was recently identified as a novel regulator of PM cholesterol and PI(4,5)P_2_ content in both cell lines (Wang and others 2019) and neurons (Weber-Boyvat and others 2022). ORP2 via its FFAT domain binds VAP, thereby inducing the formation of VAP oligomeric complexes. Recent work in murine primary neurons showed that these VAP oligomers are localized to neurites in young (DIV7) neurons but are rarely present in mature neurites. In agreement with this, loss of ORP2 increased cell death and reduced neurite length in young but not in old neurons in culture (Weber-Boyvat and others 2021), indicating a role for ORP2-VAP complexes in neurite development. Other proteins that have emerged as important players at MCSs by interacting with lipid transfer proteins are the SNARE proteins (Weber-Boyvat and others 2021). SNARE proteins are small, membrane-anchored proteins that drive membrane fusion, for instance, during exocytosis. Nonfusogenic contacts between the ER-resident SNARE protein Sec22b and syntaxin 1 on the PM have been implicated in axon development (Petkovic and others 2014). Sec22b–syntaxin 1 complexes tether the ER and the PM at a distance of about 10 nm in the axonal growth cone. ER-anchored extended-synaptotagmin 2 (E-Syt2) is also part of this complex and stabilizes axonal ER-PM contact sites (Gallo and others 2020). E-Syts contain Ca^2+^-binding C2 domains and a central synaptotagmin-like mitochondrial lipid-binding protein (SMP) domain that, once dimerized, can generate a hydrophobic tunnel for lipid binding and transfer between the ER and the PM (Schauder and others 2014). Whereas SEC22b depletion reduced axonal growth, overexpression of wild-type E-Syt, but not a lipid transfer-defective mutant, resulted in neuronal membrane expansion in the form of filopodia and hyperramified axons, an effect that depends on syntaxin 1 and Sec22b. Thus, the tripartite complex between E-Syt2, syntaxin 1, and Sec22b tethers the ER to the axonal PM and provides biomolecules needed for neuronal plasma membrane expansion (Gallo and others 2020). Although the specificity of the SMP domain is limited, E-Syts are thought to mediate the extraction of diacylglycerol (DAG) from the PM. How such specificity is achieved is unclear. In this context, it is interesting to note that loss of lipin 1, a protein that controls the switch between triglyceride and phospholipid synthesis, results in enhanced axon growth and repair in DRG neurons and retinal ganglion cells (Yang and others 2020). These data suggest that ER-mediated biosynthesis of phospholipids and their transfer to the PM are important for axon regeneration.

Classically, lipid transporters between membranes shuttle only one lipid at a time (including the ones mentioned above). Recently, however, a new superfamily of lipid transfer proteins has been described that form long, extended channels that are lined by hydrophobic residues (Fig. 4). These grooves are built from multiple repeating modules that consist of five β-sheets followed by a loop and are suitable for binding tens of lipids at once, making them very well suited for the bulk transport of lipids to promote membrane expansion. The presence (or prediction) of lipid-binding chorein motifs and/or APT1 domains places proteins in this superfamily, although these domains make up only a small portion of the hydrophobic groove. To date, this superfamily consists of VPS13 family members, ATG2, SHIP164, KIAA1109 (Tweek in *Drosophila*, Csf1 in yeast), and the Hobbit (Hob) proteins (Neuman and others 2022a, 2022b). The mechanisms of action, physiologic regulation, and functions of these large, nonvesicular lipid transporters are largely unknown, but giving their function in bulk lipid transport, they could well be playing important roles in fast-growing neurons. Interestingly, mutations in many of these proteins have been associated with neurodegenerative and neurodevelopmental disorders. The best studied are the VPS13 proteins (named after their yeast homolog Vps13p), of which humans express four homologs that are mutated in distinct neurologic disorders: chorea-acanthocytosis (VPS13A), Cohen syndrome (VPS13B), early onset Parkinson’s disease (VPS13C), and ataxia (VPS13D) (Dziurdzik and Conibear 2021) (Table 1). Consistent with a role in lipid transfer, VPS13 proteins localize to different sets of MCSs, although their large size and low expression levels in mammalian cells make it hard to study exactly what membranes they are localized to. VPS13A and VPS13C are present in ER membranes, where they partially colocalize with mitochondria. In agreement with this, VPS13A knockout (KO) mice display alterations in mitochondrial morphology in the sperm midpiece, leading to infertility (Leonzino and others 2021). Recent work shows that VPS13A can also localize to ER-PM contacts (Guillen-Samander and others 2022), but if and how these contact sites influence neuronal membrane expansion or the degeneration of striatal neurons in chorea-acanthocytosis remains unknown. Of the other superfamily proteins, ATG2, KIAA1109, and the Hob proteins also have been shown to localize to the ER. ATG2 is an autophagy factor that has been implicated in the expansion of the phagophore membrane via lipid transfer (Osawa and others 2019), while the roles of KIAA1109/Tweek and Hob proteins are more obscure. In both yeast and *Drosophila*, Hob proteins localize to ER-PM contact sites, where they may play a role in the distribution of lipids and in the modulation of regulated exocytosis in professional secretory cells, including insulin-producing cells and larval salivary glands. Hob loss of function in *Drosophila melanogaster* leads to a dramatic reduction in pupal body size (hence the name), caused by defects in endocrine signaling and release of insulin (Neuman and others 2022a). In the same study, Hob was found to bind several lipids, including phosphatidylinositol (PI), PI(4)P, PI(4,5)P_2_, and phosphatidylinositol 3,4,5-triphosphate (PI(3,4,5)P_3_), similar to the APT1 domains of VPS13 proteins. Whereas Hob proteins (KIAA0100 in humans) have not been linked to human disease yet, mutations in KIAA1109 cause Alkuraya-Kučinskas syndrome, an autosomal recessive, neurodevelopmental disorder characterized by global developmental delay and brain abnormalities associated with cerebral parenchymal underdevelopment (Kumar and others 2020). Similar to Hob proteins, KIAA1109 is found at contact sites between the ER and other organelles. The *Drosophila* ortholog of KIAA1109, Tweek, has been shown to regulate synaptic PI(4,5)P_2_ levels, vesicle recycling, and the growth of the neuromuscular junction (Khuong and others 2010; Verstreken and others 2009). In agreement with this, a recent study shows that genetic deletion of KIAA1109 in mice leads to perinatal lethality and an impaired motor innervation and neurotransmission at the neuromuscular junction (Liu and Lin 2022). In patient-derived fibroblasts carrying heterozygous KIAA1109 variants, endocytosis and endosomal trafficking are compromised (Kane and others 2019), suggesting that human KIAA1109 function might be conserved. Recently, Tweek/KIAA1109 also has been implicated in the supply of phosphatidylethanolamine (PE) to the ER, thereby supporting the biosynthesis of lipid anchors needed for many cell-surface proteins (Toulmay and others 2022). Whether KIAA1109 influences neuronal membrane growth or PM composition is unknown, nor is it clear how human KIAA1109 mutations lead to neurodevelopmental disorders.

**Table 1.** Location, Proposed Function, and Associated Neurologic Diseases of ER-Organelle MCS Proteins.

Contact Sites and Proteins	Proposed Functions	Roles in Neurons	Associated Neurologic Diseases
ER-plasma membrane			
VAP-ORP2-SNAREs	Lipid transport	Promotion of cell development and neurite outgrowth (Weber-Boyvat and others 2021)	ALS
Sec22b-Stx1-Esyt2	Lipid transport	Neurite growth (Gallo and others 2020; Petkovic and others 2014).	
STIM2-ORAI1	Ca^2+^ homeostasis	Modulation of spontaneous glutamate release by SOCE (Chanaday and others 2021)	
STIM1-unknown	Ca^2+^ homeostasis	Control of neurotransmitter release via a SOCE-independent mechanism (de Juan-Sanz and others 2017)	
VAP-Kv2.1	Ca^2+^ homeostasis	Modulation of ER Ca^2+^ uptake during neuronal activity (Panzera and others 2022)	
RyR-Kv2.1-Cav1.2	Ca^2+^ homeostasis	Clustering of Cav1.2 channels in direct apposition to ER-localized RyRs; increasing Cav1.2 activity (Vierra and others 2019)	
KIAA1109/Tweek-unknown	Lipid homeostasis	Regulation of synaptic vesicle recycling and neuromuscular junction growth (Khuong and others 2010; Verstreken and others 2009)	Alkuraya-Kučinskas syndrome
ER-mitochondria			
VAPB-PTPIP5	Ca^2+^ homeostasis	Regulation of synaptic activity (Gomez-Suaga and others 2022; Gomez-Suaga and others 2019)	Parkinson’s disease, ALS/FTD
ER-LE/Lys			
Protrudin-PDZD8	Lipid transport	Endosome maturation and maintenance of neuronal polarity (Gao and others 2022; Shirane and others 2020)	
TMEM16K-Rab7	Lipid homeostasis	Regulation of endosomal sorting and neuromuscular function (Petkovic and others 2020)	SCAR10
ER-microtubule			
STIM1-EB1/EB3	Localization of Ca^2+^ release	Regulation of microtubule and ER remodeling in steering growth cones (Pavez and others 2019)	
Different ER-MCS			
VPS13 paralogues	Lipid transport	Unknown	VPS13A: ChAc
			VPS13B: Cohen syndrome
			VPS13C: Early onset Parkinson’s disease
			VPS13D: Spastic ataxia

ALS, amyotrophic lateral sclerosis; ChAc, chorea-acanthocytosis; ER, endoplasmic reticulum; FTD, frontotemporal dementia; LE, late endosome; Lys, lysosomes; MCS, membrane contact site; SCAR10, spinocerebellar ataxia type 10; SOCE, store-operated Ca^2+^ entry.

The classical ER-shaping reticulon (Rtn) proteins Rtn3 and Rtn4 have also been implicated in processes related to PM function, including endocytosis and exocytosis (Caldieri and others 2017; Mukherjee and Levy 2019; Siddiqi and others 2018). For instance, Rtn3 and a proteolytic C-terminal fragment of Rtn4 are released in extracellular vesicles, thereby potentially regulating neurite growth and axon regeneration (Sekine and others 2020; Wojnacki and others 2020). The molecular mechanisms that underlie these effects are poorly understood. Moreover, Rtn3-dependent ER-PM contacts have been shown to promote the formation of peripheral ER tubules that facilitate epidermal growth factor receptor (EGFR) endocytosis in nonneuronal cells (Caldieri and others 2017). Whether these Rtn3-mediated contact sites also involve lipid transfer or Ca^2+^ signaling processes remains to be determined.

## ER-PM Contact Sites and Ca^2+^ Transfer in Development and Repair

Efficient axonal repair and growth not only require massive amount of lipids but also release of Ca^2+^ from intracellular stores to activate cAMP and a number of growth and regenerative processes, including degradative pathways and cytoskeleton remodeling (Petrova and others 2021). Long-range Ca^2+^ waves elicited from ER Ca^2+^ stores have been shown to play regulatory roles in neurite outgrowth and in the regenerative response to axon injury in dorsal root ganglion (DRG) neurons (Cho and others 2013). Central coordinators of cellular Ca^2+^ signals are STIM proteins (STIM1 and STIM2), Ca^2+^-sensing proteins located on the ER membrane that initiate store-operated Ca^2+^ entry (SOCE). STIM is activated upon a decrease in Ca^2+^ concentration in the ER lumen and subsequently undergoes a conformational change, oligomerizes, and relocalizes to ER-PM contact sites. Here, STIM recruits the PM Ca^2+^ channel ORAI1 to form a membrane conduit that facilitates the influx of Ca^2+^ from the extracellular space into the cytosol. STIM1-dependent SOCE is necessary for cue-induced elevations in intracellular Ca^2+^ at the axonal growth cone, thereby regulating directed growth cone motility toward Ca^2+^-dependent guidance cues such as BDNF (Mitchell and others 2012). Recent work has revealed a unique ER architecture in fast-growing, distal axons of developing primary rodent cultures, termed the ER ladder (Zamponi and others 2022). In this ladder, the rails are in proximity to the axonal PM, whereas the rungs cross perpendicularly and are in close contact with microtubules (MTs). ER “rung depletion” by Rtn2 knockdown resulted in deficient axonal trafficking of vesicular axonal cargoes and a decrease in axonal growth that might be due to the loss of interorganelle contacts or MT stability (see next paragraph). Along the ER ladder, proteins seem to be unequally distributed as STIM1 is strongly enriched at ER rungs. In agreement with its function, STIM1 relocalizes to the peripheral ER upon STIM1 activation by thapsigargin, a drug that triggers the depletion or ER Ca^2+^ stores. What exact role the ER ladder, which is absent from mature axons, plays in Ca^2+^ signaling in developing neurons is yet to be uncovered.

## ER-Mitochondria Contacts in Development, Neurotransmission, and Disease

While ER contacts with the neuronal PM seem to be an obvious route to modulate growth and development, ER-membrane contact sites with other organelles have also been implicated in neurite growth and regeneration. ER and mitochondria form specialized contacts referred to as mitochondrial-associated membranes (MAMs) that participate in the regulation of many processes such as mitochondrial division and the exchange of lipids and Ca^2+^. The main route of Ca^2+^ transfer between these organelles is facilitated by GRP75 that simultaneously interacts with the inositol 1,4,5-trisphosphate receptor (IP_3_R) in the ER and voltage-dependent anion channel 1 (VDAC1) in the outer membrane of mitochondria. Both in growing neurites and after axotomy, GRP75 protein levels increase and promote the retrograde movement of mitochondria, neurite elongation, and axon regeneration, likely by increasing Ca^2+^ transfer from the ER to mitochondria needed for local ATP generation (Lee and others 2019; Trigo and others 2019). Many other proteins are involved in the tethering and the modulation of these contact sites, including VAPB, PTPIP51, SIGMAR1, Creld, APP, Tau, Akt1, presenilins, and, possibly, α-synuclein. It seems noteworthy that all of these proteins have been implicated in neurodegenerative diseases including Parkinson’s disease and motor neuron diseases. These data suggest that ER-mitochondria signaling defects may be a common feature of neurodegenerative diseases and point to MAMs as a potential therapeutic target (Lim and others 2021; Markovinovic and others 2022; Paradis and others 2022). In support of this, synthetic linkers that increase the number of contacts between the ER and mitochondria in *Drosophila* brains have been demonstrated to extend life span and to mitigate β-amyloid toxicity in a model of Alzheimer’s disease (Garrido-Maraver and others 2020).

**Figure 5.**
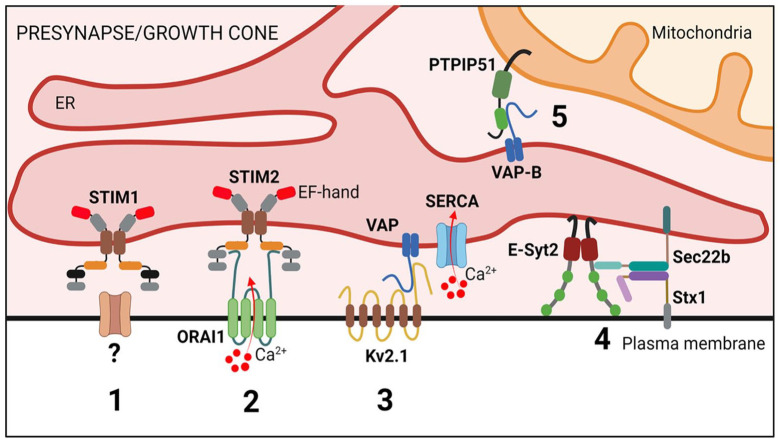
Membrane contact sites at the neuronal presynapse and in growth cones. Scheme illustrating proteins involved in endoplasmic reticulum (ER) contact sites at the neuronal presynapse and growth cone. (1) STIM1 couples with so far unknown plasma membrane protein(s) to modulate neurotransmitter release via a SOCE-independent pathway. (2) STIM2-ORAI1 contacts promote spontaneous excitatory neurotransmission, and this might be linked neurodegeneration. (3) VAP-Kv2.1 ER–plasma membrane (PM) junctions mediate ER Ca^2+^ filling at presynaptic nerve terminals, thereby modulating synaptic neurotransmission. (4) Sec22b-Stx tethers are stabilized by E-Syt2 and facilitate axon development. (5) VAP-B-PTPIP51 contacts modulate synaptic function (e.g., synaptic vesicle exocytosis). E-Syt2, extended synaptotagmin 2; Stx1, syntaxin 1; PTPIP51, protein tyrosine phosphatase interacting protein 51.

Both the ER and mitochondria are also present in synaptic regions, and signaling between these organelles likely contributes to the regulation of synaptic activity. The mitochondrial membrane protein protein tyrosine phosphatase interacting protein 51 (PTPIP51) interacts with VAP-B to form such a tether (Fig. 5). VAP-B-PTPIP51 tethers have been linked to Parkinson’s disease and to amyotrophic lateral sclerosis (ALS) as well as to frontotemporal dementia (FTD) (i.e., diseases characterized by loss of synaptic function as a key pathogenic feature). Damage to the VAP-B-PTPIP51 tethering complex has also been implicated in other neurodegenerative diseases (Gomez-Suaga and others 2022). A study using a split-GFP ER-mitochondria contact reporter (SPLICS) indicated that VAP-B-PTPIP51 contacts are present at synapses and increase in response to neuronal activity. Knockdown of either VAP-B or PTPIP51 reduces the amount of contacts and inhibits synaptic activity, including alterations in synaptic vesicle exocytosis (Gomez-Suaga and others 2019). A primary physiologic function of the VAP-B-PTPIP51 tethers is to facilitate IP_3_R-mediated delivery of Ca^2+^ from ER stores to mitochondria to increase mitochondrial ATP production. Hence, it is conceivable that the observed loss of synaptic function is an indirect consequence of defective mitochondrial ATP production in the absence of either VAP-B or PTPIP51. Additional studies are required to answer this question.

## ER and ER-PM Contact Sites in Presynaptic Neurotransmission

An important function of the axonal ER network in mature neurons is the regulation of Ca^2+^ dynamics. Ca^2+^ is central to neurotransmission and plays key roles in various forms of pre- and postsynaptic plasticity, including presynaptic short-term facilitation and depression, presynaptic long-term potentiation at hippocampal mossy fiber terminals, and the postsynaptic long-term potentiation and depression downstream of glutamate receptor activation. Conversely, neuronal activity positively regulates the mobility of the ER (Perez-Alvarez and others 2020), likely affecting the number of ER-PM contact sites (Tao-Cheng 2018). Neurotransmitter release from presynaptic terminals is primarily regulated by rapid Ca^2+^ influx through membrane-resident voltage-gated Ca^2+^ channels. Accumulating evidence further indicates that the axonal ER plays a modulatory role in synaptic transmission by regulating presynaptic and/or axonal Ca^2+^ levels. In the axon, release of Ca^2+^ from the ER and ER-Ca^2+^ depletion have been shown to increase both spontaneous and evoked release of neurotransmitters in different types of synapses (Chanaday and Kavalali 2022). The molecular pathways that underlie these highly synchronized presynaptic processes remain only partially characterized but may include ER-PM contact sites. Recent studies in hippocampal presynaptic terminals show that SOCE is mediated by STIM2, the most abundant STIM isoform in hippocampal neurons that is characterized by a lower Ca^2+^ affinity compared to its well-characterized STIM1 cousin. Chronic ER Ca^2+^ depletion and subsequent STIM2-mediated SOCE have been shown to enhance spontaneous excitatory neurotransmission (Chanaday and others 2021). In addition to the comparably slow mechanism of SOCE, neurotransmitter release may be affected by alternative modes of action of ER-PM contact sites. In a study by de Juan-Sanz and others (2017), STIM proteins were proposed to monitor decreases in axonal ER Ca^2+^ and, thereby, control neurotransmitter release via a poorly understood SOCE-independent mechanism that conceivably could involve the modulation of voltage-gated Ca^2+^ channels at the PM.

**Figure 6.**
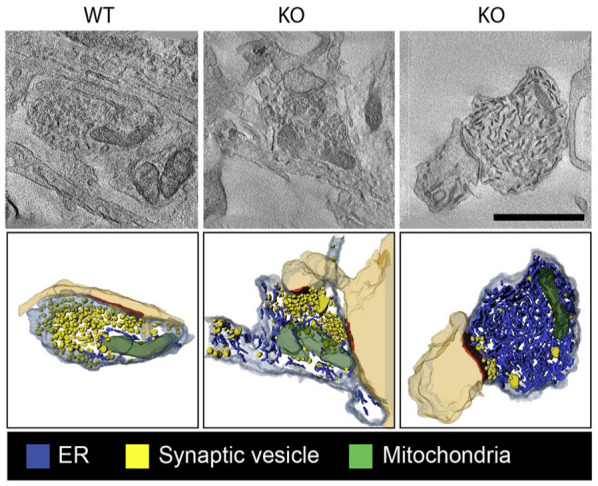
Tubular endoplasmic reticulum (ER) accumulation in autophagy-deficient axons. Single virtual sections and three-dimensional transmission electron microscopy tomography reconstructions of synaptic boutons (mouse hippocampal neurons in culture) with ER tubules in blue and synaptic vesicle (SVs) in yellow. On the left, a wild-type presynapse that contains few ER tubules is shown. In the middle and right images, neuronal autophagy is abrogated due to the absence of autophagy protein ATG5 (KO). This results in mild (middle) to severe (right) ER tubule accumulation in the axons. Adapted from Kuijpers and others (2021).

ER-PM MCSs often comprise voltage-sensitive proteins. The best-known example of this are the PM-localized voltage-dependent Ca^2+^-channel DHPR and ER-localized Ca^2+^ channel ryanodine receptors (RyRs). These proteins cooperate in muscle during excitation-contraction coupling, whereby electrical events at the PM are converted into Ca^2+^ release from the ER. In mature neurons and in the mouse hippocampus, it has recently been demonstrated that the axonal and presynaptic accumulation of RyRs in the absence of ATG5 results in increased excitatory neurotransmission as a consequence of elevated Ca^2+^ release from ER stores (Kuijpers and others 2021) (Fig. 6). In dissociated cultured hippocampal neurons, voltage-gated K^+^ (Kv) channels have been shown to associate and form clusters with ER-localized RyRs (Vierra and others 2019) and with VAP proteins (Panzera and others 2022). Kv2 channels are abundant in brain, where they play important roles in neuronal excitability. In addition, in response to ER tethering, Kv2 channels can form nonconductive clusters at the PM, where they act as specialized platforms that facilitate membrane protein trafficking and protein insertion (e.g., ion channel delivery) (Deutsch and others 2012; Vierra and others 2019). At hippocampal presynaptic terminals, VAP-Kv2.1 tethers were recently shown to position endoplasmic reticulum Ca^2+^-ATPase (SERCA) pumps nearby sources of presynaptic Ca^2+^ influx to promote synaptic transmission (Panzera and others 2022) (Fig. 5). How exactly elevated Ca^2+^ uptake by the synaptic ER during electrical stimulation modulates neurotransmission is unclear. ER-PM contact sites have further been demonstrated to facilitate presynaptic neurotransmission in *D. melanogaster* and in *Caenorhabditis elegans*. In this mechanism, the single E-Syt ortholog executes a lipid-independent role in facilitating neurotransmission, likely by regulating Ca^2+^-driven synaptic vesicle release (Kikuma and others 2017; Piggott and others 2021). Surprisingly, mice devoid of all known E-Syts are viable and fertile and do not show any overt brain abnormalities or defects in neurotransmission, possibly due to the upregulation of compensatory genes (Tremblay and Moss 2016).

While most synaptic ER-PM contacts are predicted to play roles in modulating Ca^2+^ levels, distinct MCSs might affect neurotransmission by acting as lipid transfer tethers. Recent work in hippocampal synapses indicates that ORP2 interacts with the plasma membrane SNAREs syntaxin 1A and SNAP-25, regulating synaptic cholesterol levels and neurotransmitter release (Weber-Boyvat and others 2022). Another example is the ER-localized phospholipid transporter TMEM24 that is enriched in neurons where it populates, together with Kv2.1 and VAP, ER-PM contacts. In nonneuronal insulin-secreting cells and tissues, TMEM24-mediated vectorial transfer of PI from the ER to the PM has been shown to control calcium oscillations and, thereby, pulsatile insulin secretion (Lees and others 2017). It seems tempting to speculate that TMEM24 plays similar roles in neurons, although its exact physiologic function in brain remains to be determined. Future studies will be needed to provide a more complete understanding of the exact roles of ER-PM contacts in mature axons and in neurotransmission in the brain.

## ER–Late Endosome/Lysosome Tethering in Development and Disease

Similar to ER-mitochondrial contacts, MCSs between the ER and late endosomes (LEs) or lysosomes (Lys) are linked to severe neurologic diseases and neurodegeneration, and they play essential roles in neuronal physiology. Different ER proteins have been identified to form tethers with LEs/Lys, including the aforementioned VAP and VPS13C proteins, as well as the ER transmembrane protein protrudin. The VAP-B loss-of-function point mutant P56S causes familial ALS (ALS8) by forming aggregates and promoting ER stress (Kanekura and others 2006). VAP-A and VAP-B are direct and indirect tethers at ER-LE MCSs via associating with ORP1L and STARD3 (Alpy and others 2013). The VAPs also enforce the ER retention of protrudin, which coincidentally binds to the late endosomal small GTPase Rab7a and to phosphatidylinositol 3-phosphate (PI(3)P), thereby promoting the association of kinesin 1 to mediate transport of LE/Lys to the tips of neurites to facilitate polarized neurite outgrowth (Raiborg and others 2015). The protrudin-associated ER protein PDZD8 also acts as a tether but additionally possesses lipid transfer activity. Together, these proteins mediate the transfer of lipids from the ER to LE/Lys, thereby affecting endosome fission and maturation. Mouse primary neurons depleted of protrudin or PDZD8 suffer from reduced axon length, while dendritic growth is increased (Gao and others 2022; Shirane and others 2020). In agreement with an important role for MCSs between the ER and LE/Lys, ER tubules that contact LAMP1-positive lysosomes at the preaxonal region promote kinesin 1–powered lysosome fission and their subsequent passage into the axon (Ozkan and others 2021). Surprisingly, in this study, protrudin depletion did not appear to hamper lysosome dispersion into the axon, possibly owing to the functional compensation of protrudin loss by other tethering factors or via distinct mechanisms of axonal lysosome transport (Ozkan and others 2021). ER-LE/Lys contact sites also play a role in regeneration processes. In a recent study by Petrova and others (2020), a phosphomimetic active protrudin was used to promote the regeneration of mature cortical axons in vitro via increased endosomal transport into the distal part of injured axons. Protrudin is a known binding partner of spastin (i.e., SPG4), an ER-resident protein that bridges ER-lysosome contacts via its binding partner IST1 (Allison and others 2017) and whose dysfunction is causally involved in the most common form of HSP, a group of inherited neurologic disorders with more than 80 associated genetic loci (Blackstone 2018).

A further group of ER-membrane tethers consists of members of the transmembrane 16 (TMEM16) family that in mammals comprise 10 subtypes that have been implicated in a broad range of functions. TMEM16K is an ER lipid scramblase, causative for a progressive neurodegenerative disease named spinocerebellar ataxia (SCAR10). Neuron-specific TMEM16K knockout mice have a progressive impairment of neuromuscular function, resembling the human disease. In primary mouse embryonic fibroblasts, ER-localized TMEM16K forms MCSs with endosomes, and TMEM16K depletion results in endosomal sorting and acidification defects (Petkovic and others 2020). Collectively, these data suggest that multiple tethers contribute to axon growth and regeneration via MCS formation between the ER and LE/Lys.

## ER-Microtubule Contacts in Development and Neurodegeneration

The correct positioning and remodeling of the ER, like that of other organelles, is mediated by motor protein-driven intracellular transport along the microtubule and actin cytoskeleton. A recent study shows that during development, the recruitment of ER tubules into one minor neurite initiates axon formation. This is put in motion through the direct binding of the ER protein P180 to the MT lattice, thereby stabilizing both ER tubules and MTs in the prospective axon (Farias and others 2019). The spatiotemporal regulation of MT abundance and properties plays a key role in axon specification and development, providing mechanical support and rails for axonal transport. A recent cryo-EM study revealed that ER and microtubules are enriched and intertwined at developing axon branch points (Nedozralova and others 2022), where they may cooperate to modulate axon branching in a way similar to their function in axon outgrowth. In *C. elegans* neurons, retraction of the ER from distal dendrites due to loss of atlastin 1 leads to the destabilization of MTs, illustrating the important role of the ER network dynamics in the cellular distribution and remodeling of MTs (Liu and others 2019). This effect might causally underlie HSP and HSAN (i.e., neurodegenerative diseases linked to the loss of atlastin 3) (Kornak and others 2014). Recent work has further indicated Ca^2+^-independent but MT-dependent roles of STIM1 in growth cone navigation. Here, the asymmetric activation of STIM1 by guidance cues induces its interaction with the MT plus-end binding proteins EB1/EB3, leading to asymmetric MT invasion and growth cones turning (Pavez and others 2019).

## Outlook

Our understanding of the roles of the ER and ER-based MCSs in neuronal development, function, and disease is still in its infancy. The identification and functional characterization of ER-based MCSs remain technically challenging, often involving multiple complementary approaches, including super-resolution light and electron microscopy, as well as proximity-based visual (e.g., split GFP, FRET, proximity ligation) and biochemical (e.g., APEX and TurboID) approaches (Kim and others 2022). Moreover, as MCSs are formed by specific pairing of proteins residing in different compartments, loss-of-function studies often fail to discern MCS-related phenotypes from others arising from protein function within a given organelle. Furthermore, MCSs are involved in the transport of small-molecule metabolites, including lipids or ions (Wu and others 2018), which often pose special analytical challenges, in particular when the nervous system is concerned. A thorough understanding of neurologic diseases caused by dysfunction of the ER and/or its contacts with other organelles (Fowler and others 2019; Kim and others 2022) will thus require the development of improved methodology to sensitively detect local alterations in metabolite, lipid, or ion concentrations, as well as new techniques that enable the direct visualization of MCSs in situ, for example, by live correlative super-resolution and electron microscopy (live-CLEM).

Many questions remain regarding the precise composition and function of the ER and its contacts with other organelles in the brain as well as its role in neurologic diseases (Fowler and others 2019; Kim and others 2022; Pallotta and others 2019). For example, at present, it is unclear whether all ER and its MCSs are compositionally and functionally equal throughout the neuronal soma, dendrites, and axon. We also know little about the dynamics and regulation of the ER and ER-based MCSs in response to neuronal activity, metabolic challenge, aging, or other types of stresses that neurons may encounter during the lifetime of an organism. It is further conceivable, if not likely, that ER-based MCSs may exhibit cell-type (i.e., neuron-subtype) specific differences with respect to composition and function. For example, multiple lines of evidence suggest a close link between Parkinson’s disease and mitochondrial dysfunction in dopaminergic neurons, possibly owing in part to defective MCSs between multiple organelles, including mitochondria, the ER, LE/Lys, and lipid droplets. Disruption of ER-mitochondria tethering and signaling has also been observed in C9orf72-associated ALS and FTD (Gomez-Suaga and others 2022). Equally strong ties exist between Alzheimer’s disease and lysosomal dysfunction in cortical neurons, in which endolysosomal abnormalities may spread to other organelles via MCSs. Cell- or neuron-subtype specific differences might conceivably result from the differential expression of proteins involved in the formation of ER-based MCSs, distinct regulation of neuronal activity and/or local Ca^2+^ homeostasis, or the vastly different morphologic architecture of distinct types of neurons, including dendritic and axonal arborization as well as overall size and, therefore, the distance between the soma and synapses. How exactly such differences lead to the characteristic features of neurologic diseases and their manifestations in specific areas of the central or peripheral nervous systems remains an area of utmost importance for the development of novel therapeutics.
